# Phosphorus deficiencies invoke optimal allocation of exoenzymes by ectomycorrhizas

**DOI:** 10.1038/s41396-020-00864-z

**Published:** 2021-01-08

**Authors:** Justin A. Meeds, J. Marty Kranabetter, Ieva Zigg, Dave Dunn, François Miros, Paul Shipley, Melanie D. Jones

**Affiliations:** 1 Biology Department, University of British Columbia, Okanagan Campus 1177 Research Road, Kelowna, BC V4V 1V7 Canada; 2 British Columbia Ministry of Forests, Lands and Natural Resource Operations, P.O. Box 9536, Stn Prov Govt, Victoria, BC V8W 9C4 Canada; 3 Chemistry Department, University of British Columbia, Okanagan Campus 3187 University Way, Kelowna, BC V4V 1V7 Canada; 4 Natural Resources Canada, Pacific Forestry Centre, 506 Burnside Road West, Victoria, BC V8Z 1M5 Canada

**Keywords:** Ecology, Microbial ecology

## Abstract

Ectomycorrhizal (EM) fungi can acquire phosphorus (P) through the production of extracellular hydrolytic enzymes (exoenzymes), but it is unclear as to the manner and extent native EM fungal communities respond to declining soil P availability. We examined the activity of six exoenzymes (xylosidase, *N*-acetyl glucosaminidase, β-glucosidase, acid phosphomonoesterase, acid phosphodiesterase [APD], laccase) from EM roots of *Pseudotsuga menzesii* across a soil podzolization gradient of coastal British Columbia. We found that APD activity increased fourfold in a curvilinear association with declining inorganic P. Exoenzyme activity was not related to organic P content, but at a finer resolution using ^31^P-NMR, there was a strong positive relationship between APD activity and the ratio of phosphodiesters to orthophosphate of surface organic horizons (forest floors). Substantial increases (two- to fivefold) in most exoenzymes were aligned with declining foliar P concentrations of *P*. *menzesii*, but responses were statistically better in relation to foliar nitrogen (N):P ratios. EM fungal species with consistently high production of key exoenzymes were exclusive to Podzol plots. Phosphorus deficiencies in relation to N limitations may provide the best predictor of exoenzyme investment, reflecting an optimal allocation strategy for EM fungi. Resource constraints contribute to species turnover and the assembly of distinct, well-adapted EM fungal communities.

## Introduction

Phosphorus (P) availability can be a fundamental constraint to primary productivity in many ecosystems, especially older soils where total P typically decreases and the ratio of organic P (P_o_) to inorganic P (P_i_) increases [1]. P-depleted soils can also be found with podzolization under wetter climates, where precipitation encourages the solubilization of primary mineral P, accelerates conversion of P_i_ to P_o_ by microorganisms, and increases the formation of secondary mineral P, thereby reducing the availability of orthophosphate for uptake by roots and microbes [2]. P deficiencies are also starting to occur in European and North American temperate forests affected by N pollution and climate change [3, 4].

Members of the Pinaceae are obligately symbiotic with ectomycorrhizal (EM) fungi [5]. In these trees, virtually all of the fine absorptive roots form ectomycorrhizas; hence, the EM roots can be considered the nutrient-absorbing organs of these plants and the ability of the fungi to absorb nutrients will influence plant nutrient status [6–8]. EM fungi use several strategies to enhance mobilization of both inorganic and organic P [9–11]. Of particular importance for P mobilization is extracellular hydrolytic enzymes (exoenzymes) that adhere to the surfaces of mantle and extramatrical (i.e., external to the mantle) hyphae of ectomycorrhizas [8, 12–14]. Two key types of exoenzymes involved in the direct hydrolysis of P from phosphoesters of soil organic matter are acid phosphomonoesterases (APMs) and phosphodiesterases [15, 16]. While phosphodiesterase activities of ectomycorrhizas have been little studied, increases in phosphomonoesterase activity of litter colonized by EM fungi have been associated with loss of P from the litter and accumulation of P in EM seedlings [17, 18]. Other fungal exoenzymes, such as cellobiohydrolase, β-glucosidase (GU), β-glucuronidase, xylosidase (X), and laccase (LAC), play an indirect role in P acquisition by degrading cell walls in plant and fungal necromass, thereby providing access to the nutrients within [8, 19]. EM fungal species differ considerably in their expression of these enzymatic P-acquisition traits [20–22]; consequently, the species and diversity of EM fungi colonizing a plant influences its P content (e.g., [6, 23]).

EM fungal communities are highly diverse, with community composition filtered by deterministic processes (host and soil environment), along with some stochastic (dispersal) influences [24–26]. Evidence is accumulating that compatibility between soil chemistry and EM fungal nutrient acquisition traits, including extracellular enzyme activities and ion flux rates, is part of the environmental filter for EM fungal community assembly. This has been observed in forests with different host species [27], and over successional [28, 29] and nitrogen (N) gradients [30]. Such congruence between functional traits and local edaphic conditions can arise through several mechanisms: species selection, adaptation of fungal populations to site conditions over time, or phenotypic plasticity (i.e., acclimation; [27]). Site-specific EM fungal communities possessing traits that increase access to growth-limiting nutrients are expected to contribute to the overall fitness of the plant partner and, hence, the symbiosis [31]. Indeed, the ability of seedling genotypes to survive on a specific soil is mediated, at least in part, by being able to associate with the EM fungi adapted to those soils [32]. If tree genotypes are not able to associate with the locally adapted EM fungi, their growth may be impaired relative to local tree genotypes [33]. As more forest regions become P-limited, the availability of EM fungal communities adapted to accessing specific form of soil P will be important for survival and/or migration of trees; however, little is known about the natural variation in P-acquisition strategies exhibited by EM fungal communities as sources of P change across landscapes.

Although we have focussed on response to soil P levels alone, a potentially more comprehensive evaluation of EM fungal response to nutrient deficiencies would be through resource stoichiometry, particularly the simultaneous constraints of N and P (expressed as an N:P ratio) on metabolism and growth [34]. The balance of N and P limitations is particularly relevant to the principle of optimal allocation, which suggests that mycorrhizal fungi will preferentially allocate energy toward acquiring the resources that are most limited in supply [31, 35, 36]. This principle has been widely established experimentally for decomposer biota such as bacteria and saprotrophic fungi [37]. For example, microbes will maximize N-acquiring enzymes under low N:P resources such as leaf litter with limited N mineralization rates, but then shift to P-acquiring enzymes under high N:P litter where N supply is less constraining [38]. The sensitivity of exoenzyme production to both N and P supply, rather than P alone, could therefore broaden our perspectives on stand nutrition and the rationale behind nutrient acquisition strategies of mycorrhizal fungi.

Here we report on P-acquisition exoenzyme activities associated with ectomycorrhizas along a podzolization gradient of coastal forests across Vancouver Island, where there has been a considerable depletion of orthophosphate and primary mineral P, alongside an accumulation of P_o_ [7]. Our goal was to test whether EM fungal P-acquisition strategies changed along this soil P gradient, while holding host tree species and tree age constant. We measured the activity of a series of exoenzymes found on the surfaces of EM root tips of coastal Douglas-fir (*Pseudotsuga menzesii var. menzesii*) stands and relate the exoenzyme concentrations to soil P, including inorganic P as well as organic fractions revealed by ^31^P NMR. We hypothesized that activities of all exoenzymes would be higher in strongly podzolized soils, where P_o_ contributes the majority of total P. In addition, we tested whether exoenzyme activities were predominantly related to soil P availability or whether a combined index with N (as soil or host N:P) would better reflect allocation strategies by EM fungi. Last, we explored whether the highest levels of phosphatase activities would be associated with EM fungal species restricted in their distribution to the podzolized, low-P soils. Results of our study will provide further insights into the manner and extent EM fungal communities of a single tree host have responded to declining soil P availability during soil development.

## Methods

### Site descriptions

We selected ten locations across an ~100-km extent of southern Vancouver Island (British Columbia, Canada) that encompassed a podzolization gradient driven by an orographic rainshadow, as described in ref. [7]. The sites were selected to capture a gradient in soil N and P availability, ranging from upland mesotrophic stands in both dry (referred to as “Upland Brunisol,” *N* = 4) and wet (“Upland Podzol,” *N* = 4) coastal forests, along with moist, nutrient-rich soils in wet coastal forests (“Lowland Podzol,” *N* = 4). We established one plot (25 × 25 m in size) at each site, with the exception of Branch 167 and 247, where the complex topography enabled us to sample both an Upland and Lowland Podzol plot (~400 m apart at both sites). All locations had second-growth *P*. *menzesii* stands that ranged in age from 40 to 60 years.

### Soil and foliar attributes

Soil and foliar data were first reported in ref. [7], with the additions here of Bray P and ^31^P NMR spectroscopy conducted later on the same soil samples. Total soil P (P_t_) was the sum of inorganic (P_i_) and organic phosphorus (P_o_) concentrations as determined by the ignition method using a sulfuric acid (0.5 M) digest [39]. P in soil solution, or weakly adsorbed to soil colloids, was determined by a Bray P extraction [39]. One representative forest floor sample (the partly and well-decomposed horizons of the surface organic layer [40]) from two plots of each soil type (Upland Brunisol, Upland Podzol, Lowland Podzol) was selected for ^31^P NMR analysis following [41]. Only forest floor samples were analyzed because mineral soil samples did not have high enough P concentrations to detect peaks. Briefly, samples were shaken in 0.5-M NaOH and 0.1-M disodium EDTA solution for 4 h, centrifuged, filtered to 6 µm, freeze-dried, and re-dissolved in minimum amount of deuterated solvent. Dimethyl methyl phosphonic acid was used as an internal standard. Proton-decoupled spectra were obtained on a Varian 400-MHz spectrometer and processed using MestreNova v12.0 software (Supplementary Method 1).

### Sampling of EM root tips

Sampling of EM roots took place over 3 weeks, from late May to mid-June 2018. At the start of each week, we sampled four plots that included one or two replicates of each soil type. Seven soil cores, ~15-cm wide and 15-cm deep, were collected randomly from each plot using a long knife and pruning clippers. The soil cores included both forest floor and mineral soil substrates, and many EM roots were found near the interface of these two substrates. Soil cores were transported on ice to the lab for overnight storage at 4 °C. Exoenzyme assays took place over the subsequent 3 days of each sample week.

### Microplate exoenzyme activity assays

Exoenzyme activity assays (Supplementary Method 2) were modified from protocols of refs. [20, 42, 43] and the Kennedy lab website (https://cbs.umn.edu/kennedy-lab/protocols). Starting the day after field sampling, the soil cores of each plot were examined and fresh, vigorous clusters of EM root tips of the same morphology were selected and excised to proceed with exoenzyme activity assays. Distinct EM morphotypes were taken from each soil sample to ensure a wide representation of EM fungal species per plot, although some common morphotypes (e.g., *Cenococcum*) were selected from multiple soil samples within a plot. We retrieved between 10 and 16 morphotype clusters per plot, for a total of 157 clusters across all 12 plots.

Surface debris and soil particles were carefully removed from the EM root tips under a dissecting scope. Emanating hyphae were kept intact as much as possible during cleaning for those mantles with short-, medium- and long-distance exploration types [44]. Five individual EM root tips from each cluster were removed for the exoenzyme assay. The exoenzyme activities were assayed in series on each root tip (rinsing the roots between assays) in the following order: xylosidase (X), *N*-acetyl glucosaminidase (NAG), β-GU, APM, acid phosphodiesterase (APD), and LAC. The exoenzymes can be categorized as nutrient acquisition enzymes (NAG, APM, and APD), which hydrolytically cleave the target nutrient from the organic substrate, and lignocellulosic enzymes (X, GU, and LAC), which act to break down plant and fungal cell walls, in turn releasing the cellular contents for further action of nutrient acquisition enzymes. All the exoenzymes assayed are known to be cell-wall bound [45], and thus, their activities are not dependent on a continued supply of fixed carbon from the plant. The activities recorded in the lab should be considered as an index of activities present at the time of sample collection, including those of microbial communities living on the surface of the mycorrhizal tips. The APD assay was modified from ref. [43] for use on root tips rather than soil, with volumes for all reagents adjusted for 96-well microplates. Following the fluorescence or absorbance assays, the root tips were then transferred into a clear 96-well microplate and scanned (Epson V800) for an estimate of projected area using WinRhizo software (Regent Instruments Inc., Québec, Canada) (Supplementary Method 2).

### Molecular analysis

For each cluster that was assayed for exoenzymatic activity, 5–10 fresh EM tips were retained at −80 °C for amplification of fungal DNA following a CTAB buffer extraction protocol (Supplementary Method 3). Fungal DNA was amplified using the universal ITS5/ITS4 or the basidiomycete-specific ITS1F/LR21 primer pairs. Forward and reverse sequences were aligned using Sequencher 4.7 (Gene Codes Corporation, Ann Arbor, MI USA). Sequences were BLAST-searched through the UNITE sequence database to assign Species Hypothesis based upon a >98.5% similarity criteria [46].

### Data analysis

Prior to analysis, the exoenzymatic activities for all five root tips per cluster were averaged. Soil nutrient content (0–20-cm mineral soil plus forest floor) was determined using bulk density, coarse fragment content, and forest floor depths as outlined in ref. [7]. Relationships between exoenzymatic activities and soil P characteristics (P_i_, P_o_, and Bray P as kg ha^−1^), soil C:N (molar), soil N:P_t_ (molar), foliar N (%), foliar P (%), and foliar N:P ratio (molar) were evaluated by linear regression using lm() function in R 3.4.1 [47]. We used all the exoenzymatic data in the analysis, regardless of whether the fungal partner was identified molecularly, as we considered morphological classification sufficient to determine mycorrhization. Before regressions were conducted, exoenzymatic activities were averaged by plot and then log-transformed in order to meet the normality criteria for linear regressions. Regression results for P_i_ and Bray P were virtually identical so for brevity only P_i_ outputs are presented here. A nonmetric multidimensional (NMDS) ordination of community composition between the three soil types, based on species incidence (Bray–Curtis dissimilarity), was undertaken with metaMDS() function and function ggplot2() [47].

## Results

### Soil P status

Upland Brunisols were well-endowed with readily available P, as indicated by Bray P concentrations ~25 times greater (average 83 [SE 14] mg kg^−1^ in mineral soil) than Podzols (average 3.2 [SE 0.6] mg kg^−1^; Table 1). A similar contrast was found with inorganic P (P_i_) concentrations, which averaged 760 [SE 101] mg kg^−1^ in mineral soil for Brunisols and only 98 [SE 23] mg kg^−1^ across Podzol plots. Organic P concentrations (P_o_), by contrast, were generally highest across lowland Podzols, especially in mineral soils and forest floors (Table 1). Data on the P content (kg ha^−1^) of the upper profiles of these soils were reported previously, along with foliar N% and P% [7].

**Table 1:** Average soil phosphorus concentrations of forest floor and mineral soil for each plot, including Bray P, inorganic P (P_i_) and organic P (P_o_) (SE in brackets).

Soil type		Plot	Forest floor			Mineral soil		
			Bray P (mg kg^−1^)	P_i_ (mg kg^−1^)	P_o_ (mg kg^−1^)	Bray P (mg kg^−1^)	P_i_ (mg kg^−1^)	P_o_ (mg kg^−1^)
Upland Brunisol	Salt Spring		92.6	279	890	53.5 (3)	621 (38)	179 (34)
	Niagara		73.2 (2)	211 (20)	825 (30)	91.6 (15)	959 (28)	177 (17)
	Sooke		66.4	262	593	68.6 (10)	555 (138)	138 (60)
	Mt Prevost		–	–	–	118.4 (22)	903 (126)	233 (25)
Upland Podzol	WC 1000		19.5 (1.0)	97 (8)	617 (72)	1.7 (0.3)	46 (14)	163 (46)
	Fairy Lake		20.0 (0.8)	104 (14)	549 (18)	2.3 (0.4)	51 (2)	205 (14)
	Br. 247-59		24.1 (2.3)	140 (22)	554 (11)	2.4 (0.5)	31 (9)	134 (11)
	Br.167-126		14.9 (2.3)	103 (11)	401 (34)	2.3 (0.4)	235 (23)	290 (15)
Lowland Podzol	Br. 136		15.3 (1.7)	132 (13)	676 (119)	3.1 (0.1)	103 (18)	401 (29)
	Br. 247-63		16.5 (0.3)	59 (17)	729 (297)	6.2 (1.5)	136 (10)	416 (80)
	Klanawa		19.6 (0.7)	132 (9)	2256 (377)	5.1 (1.5)	89 (35)	535 (35)
	Br. 167-105		18.1 (0.8)	128 (4)	1860 (113)	2.6 (0.8)	93 (11)	513 (18)

Note some limited forest floor subsampling in Brunisols due to thin depths.

In addition to differences in total P_o_, NMR spectra revealed considerable differences in the chemical make-up of the organic P among soil types (Supplementary Fig. 1). Podzols had approximately twofold higher phosphodiester to orthophosphate ratios when compared to Brunisols, while phosphomonoester ratios were lowest in Upland Podzols and highest in Lowland Podzols (Table 2). Phosphonates were reasonably abundant in Lowland Podzols but were not detected in Brunisols (Table 2).

**Table 2:** Integration values for all NMR peaks when orthophosphate was set to 1.00.

Soil type	Plot	Monoesters	Diesters	Pyro-phosphate	Poly-phosphate	Phosphonates	P_total_^NMR^	Orthophosphate/total
Brunisol	Sooke	0.82	0.19	0.16	0.15	ND	2.32	0.43
Brunisol	Niagara	1.03	0.28	0.17	0.22	ND	2.70	0.37
Upland Podzol	Fairy Lake	0.61	0.42	0.16	0.24	0.07	2.50	0.40
Upland Podzol	WC 1000	0.65	0.48	0.14	0.23	0.08	2.58	0.39
Lowland Podzol	Branch 136	1.30	0.48	0.18	0.19	0.32	3.47	0.29
Lowland Podzol	Branch 247-63	1.45	0.51	0.18	0.20	0.28	3.62	0.28

The values are proportional to P concentration. P_total_^NMR^ is the sum of all integration values except the internal standard.

*ND* not detected.

### Exoenzyme activity in relation to soil nutrient status

**Fig. 1:**
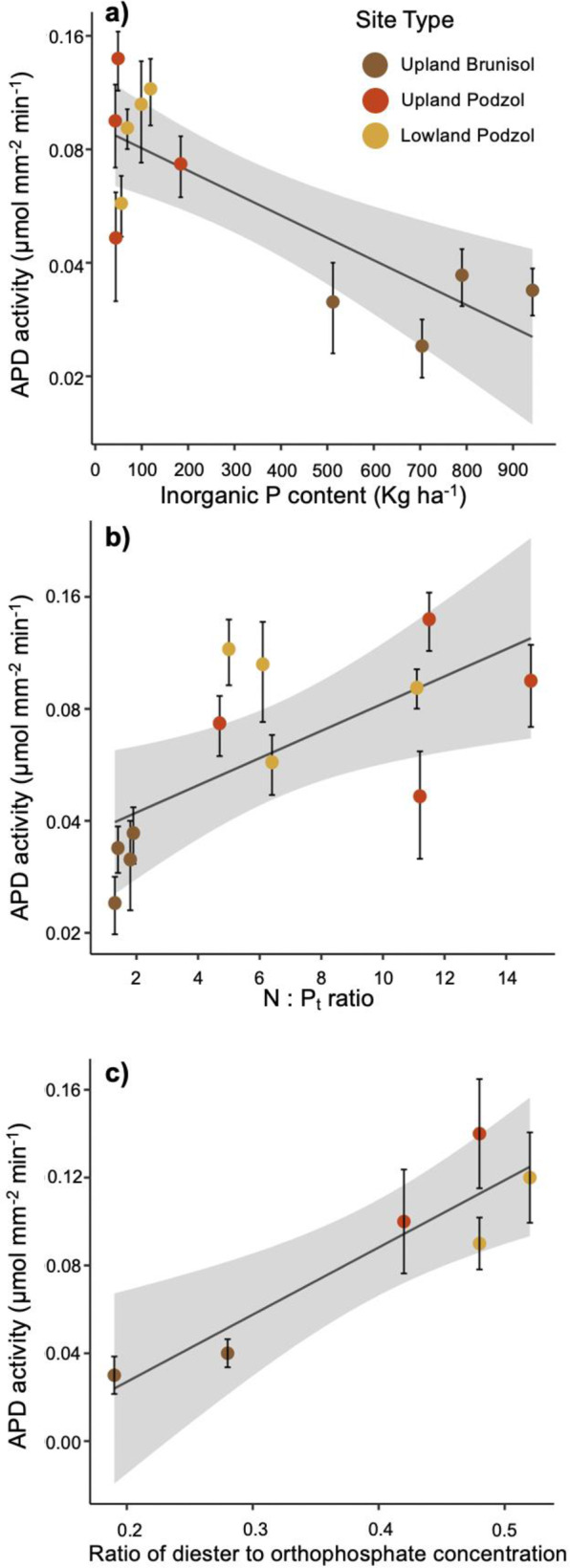
Linear regressions between average acid phosphodiesterase (APD) activity (log-transformed) and soil properties. APD activity in relation to **a** soil inorganic P content, **b** soil N:Pt molar ratio (*N* = 12), and **c** ratio of diester to orthophosphate concentration (*N* = 6). (SE of plots as error bars, SE of regressions as shaded area).

Contrary to our hypothesis, none of the six exoenzyme activities measured on EM root-tip surfaces was related to soil P_o_ content (kg ha^−1^ of the upper profile; Table 3). However, APD activity had a highly significant (*p* = 0.002, adj. *R*^2^ = 0.59), negative association with P_i_ content (Fig. 1a), along with a similar but more marginal decline in APM activity with increasing P_i_ (Table 3). The relationship of APD activity to soil P_i_ was logarithmic, with a marked increase at P_i_ contents <150 kg ha^−1^ (equivalent to <5 kg ha^−1^ Bray P), for a fourfold increase (0.03–0.12 μmol mm^−2^ min^−1^) in activity along the gradient from Brunisols to Podzols (Fig. 1a). The corresponding increase in APM activity was ~2.5-fold (0.15–0.35 μmol mm^−2^ min^−1^), although the concentrations of APM were 3–4× greater than APD overall. There were no significant changes in exoenzyme activity with soil N availability (as soil C:N ratio) over the podzolization gradient, but APD and β-GU showed a positive trend with soil N:P_t_ (Table 3 and Fig. 1b).

**Table 3:** Statistical significance of exoenzyme activities (μmol mm^−2^ min^−1^) in linear regressions with soil inorganic P (P_i_) and organic P (P_o_) content (kg ha^−1^), along with soil C:N and N:P_t_ ratios (enzymes values log-transformed, *p* values < 0.05 in bold).

	Soil P_i_			Soil P_o_			Soil C:N			Soil N:P_t_		
	*p* value	Slope	*R*^2^	*p* value	Slope	*R*^2^	*p* value	Slope	*R*^2^	*p* value	Slope	*R*^2^
APM	0.068	−4.0e^−4^	0.22	0.477	–	–	0.142	–	–	0.123	–	–
APD	**0.002**	**−1.4e**^**−3**^	**0.59**	0.165	–	–	0.331	–	–	**0.017**	**3.8e**^**−2**^	**0.40**
LAC	0.118	–	–	0.355	–	–	0.100	–	–	0.130	–	–
NAG	0.472	–	–	0.502	–	–	0.483	–	–	0.264	–	–
GU	0.101	–	–	0.240	–	–	0.544	–	–	0.062	1.4e^−2^	0.24
X	0.186	–	–	0.195	–	–	0.379	–	–	0.102	–	–

*APM* acid phosphomonoesterase, *APD* acid phosphodiesterase, *LAC* laccase, *NAG* N-acetyl glucosaminidase, *GU* β-glucosidase, *X* xylosidase.

While exoenzyme activity displayed no significant relationships with total soil P_o_ content, specific components of the organic P identified by NMR appeared to be important. Specifically, we noted a strong, positive relationship (*p* = 0.002, adj. *R*^2^ = 0.90) between APD activity and the ratio of phosphodiesters to orthophosphate across the subset of sites analyzed (Fig. 1c). The relationship between APM activity and the relative concentration of phosphodiesters was also significant but weaker (data not shown; *p* = 0.048; adj. *R*^2^ = 0.58).

### Exoenzyme activity in relation to foliar nutrient concentrations and stoichiometry

**Fig. 2:**
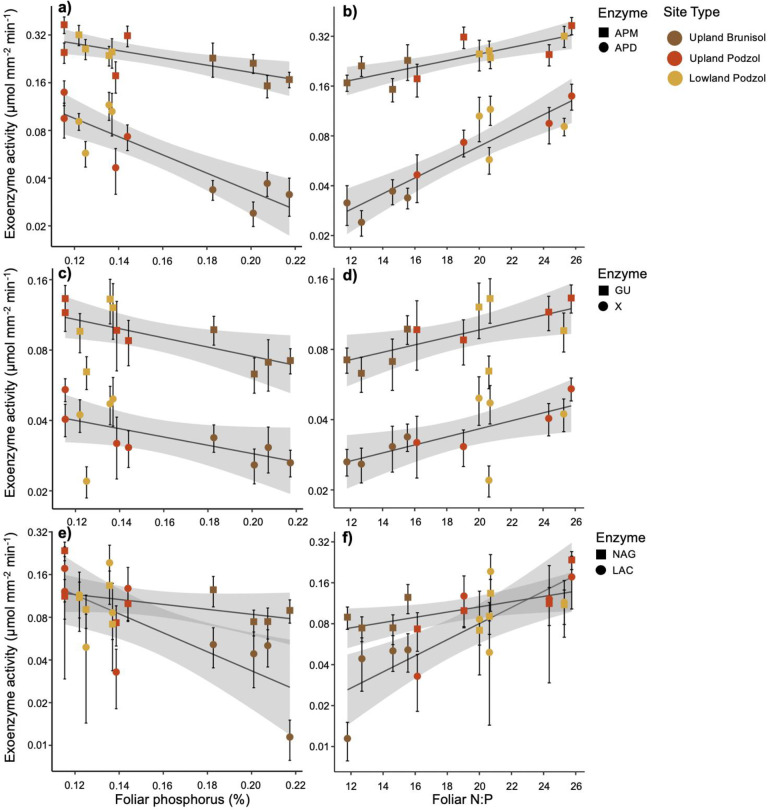
Linear regressions of exoenzyme activity (log-transformed) with foliar attributes. First column depicts foliar P% and second column foliar N:P (molar ratio) for **a**, **b** acid phosphomonoesterase (APM) and phosphodiesterase (APD); **c**, **d** xylosidase (X) and b-glucosidase (GU); and **e**, **f** laccace (LAC) and N-acetylglucosaminidase (NAG). (*N* = 12, SE of plots as error bars, SE of regressions as shaded area).

Foliar attributes, particularly P% and N:P ratio, had stronger relationships than the soil variables with exoenzyme activities (Table 4). We found clear declines in four of the six exoenzyme activities with increasing foliar P, but only one marginal gain (LAC) in relation to foliar N (Table 4 and Fig. 2). The extent of P deficiencies in relation to N, as measured by foliar N:P, was a more precise predictor than P% of exoenzyme activities, with significant positive correlations for all six exoenzymes. The relationships for foliar N:P were particularly robust (adj. *R*^2^ > 0.5) for APD, APM, and LAC activity (Table 4 and Fig. 2b, f). When aligned with increasing foliar N:P, the net gains in LAC, β-GU, and X exoenzyme activity ranged from two- to fivefold (Fig. 2d, f).

**Table 4:** Statistical significance of exoenzyme activities (μmol mm^−2^ min^−1^) in linear regressions with foliar nitrogen, phosphorus, and N:P ratio (enzyme values log-transformed, *p* values < 0.05 in bold).

	Foliar N (%)			Foliar P (%)			Foliar N:P		
	*p* value	Slope	*R*^2^	*p* value	Slope	*R*^2^	*p* value	Slope	*R*^2^
APM	0.144	–	–	**0.010**	**−1.23**	**0.45**	**0.002**	**0.010**	**0.62**
APD	0.160	–	–	**0.002**	**−0.79**	**0.58**	**<0.001**	**0.007**	**0.73**
LAC	0.078	0.28	0.21	**0.021**	**−1.01**	**0.37**	**0.004**	**0.009**	**0.54**
NAG	0.158	–	–	0.172	–	–	**0.043**	**0.006**	**0.28**
GU	0.345	–	–	**0.027**	**−0.42**	**0.34**	**0.021**	**0.003**	**0.37**
X	0.101	–	–	0.055	−0.15	0.25	**0.013**	**0.001**	**0.42**

Enzyme abbreviations as in Table 3.

### Exoenzyme activity in relation to EM fungal species

**Fig. 3:**
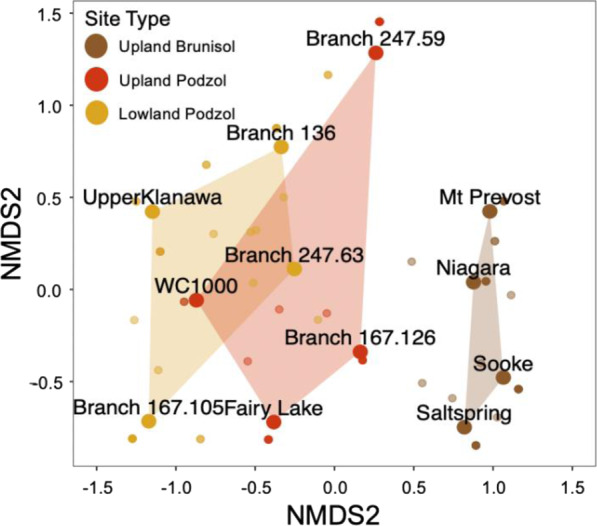
Nonmetric multidimensional scaling ordination of EM communities (species incidence) between the three soil types. Ordination of EM communities from 12 plots, individual EM species represented by smaller points colored according to predominant soil type.

Root tips examined from the 12 plots were virtually 100% EM. Of 157 EM fungal clusters sampled, 130 were successfully sequenced (83%). Overall, we identified 31 EM fungal species across the Brunisol plots, 36 species in Upland Podzols, and 35 species on Lowland Podzols (Supplementary Table 1). The EM fungal communities of the Podzol and Brunisol soil types clustered separately in the NMDS ordination, with some overlap between Upland and Lowland Podzol plots (Fig. 3).

**Fig. 4:**
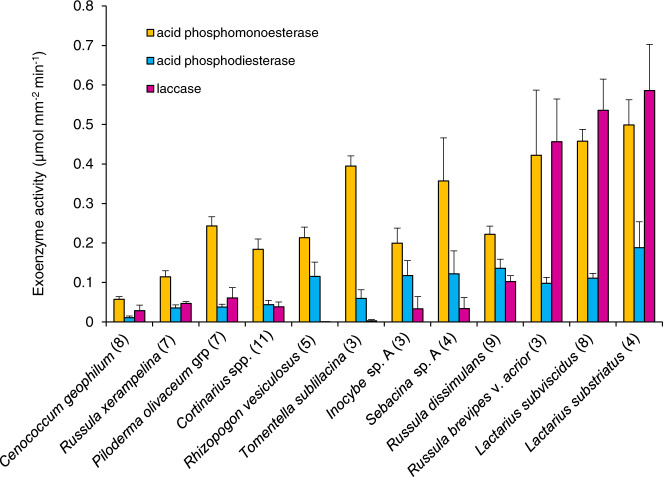
Average exoenzyme activity of acid phosphomonoesterase, acid phosphodiesterase, and laccase for select fungal taxa. Number of observations per fungal taxa in brackets, SE as bars.

A small subset of species, or multiple species from diverse genera (e.g., *Cortinarius*), was sampled frequently enough (>3) to illustrate some contrasts in exoenzyme activity among ectomycorrhizae formed by different fungal taxa (Fig. 4). The abundant ectomycorrhizae found only on Podzol sites, such as those formed by *Lactarius substriatus*, *L. subviscidus*, and *Russula brevipes* var. *acrior* had notably high expression of all three key exoenzymes (APM, APD, LAC). By contrast, *Russula xerampelina*, *Cenococcum geophilum*, and *Piloderma olivaceum* mycorrhizae, which were found almost exclusively on Brunisols, had much lower APD and LAC activity, while some genera, such as *Cortinarius*, were found across much of the edaphic gradient but featured little capacity for P-acquiring exoenzymes (Fig. 4).

## Discussion

The diversity of nutrient acquisition traits among EM fungal species has been posited to explain niche breadths of EM plants [48]. One challenging niche for the growth and establishment of trees is P-deficient soil, which can occur as a result of natural weathering as well as in landscapes where P imbalances have been induced by N deposition or climate change [6, 31]. In a naturally occurring gradient of P availability across southern Vancouver Island, we found evidence that EM fungal communities use different P-acquisition strategies depending on soil type, and that those strategies are tightly coupled with host foliar nutrition and stoichiometry. Specifically, EM fungal communities on Douglas-fir roots in P-deficient podzolized soils had much higher (two- to fivefold) activities of direct (APM, APD) and indirect (LAC, X, β-GU) P-acquiring exoenzymes than EM fungal communities in P-sufficient Brunisols. The large increases in exoenzyme activity by site-specific EM fungal communities and their associated microbiome [49] emphasizes the strong plant-soil feedbacks operating in these coastal ecosystems.

Even though phosphatase activities were highest in Podzols, with their high P_o_:P_i_ ratios, activities were more closely related to measures of inorganic than organic P in soil. This is consistent with other studies on ectomycorrhizas, where a negative relationship between inorganic P and EM mantle surface APM has been found ([50, 51], but see ref. [52]), indicating the likelihood of product (i.e., orthophosphate) inhibition [51]. Furthermore, in studies where P_o_ has been applied to pure fungal cultures, soils, or EM plants [53–56], only one study found any relationship between P_o_ and APM activity, in that case a negative one [57]. The lack of any relationship between phosphatases and total soil P_o_ in our study and others may be because some forms of the organic P do not act as a substrate for these exoenzymes. For example, phosphonates, which were relatively abundant in the lowland Podzols, are generally considered very resistant to microbial degradation [58]. In addition, a large proportion of the P_o_ was likely phytate [59], which, although it would have been grouped with monoesters in our NMR analysis, may not be used effectively by EM fungi [60]. When we quantified the relative amounts of diesters using ^31^P NMR, we were able to show a strong positive correlation with both APD and APM, suggesting that these exoenzymes were being produced in response to the increase of their substrates within the soil organic matter at Podzol sites. Phosphomonoesters are one of the products of phosphodiesterase activity.

Of the two types of phosphatase activity measured, APD appeared to be especially important in the response of ectomycorrhizas to low P_i_. While the production of APD has been described as an important P-acquisition strategy of mycorrhizal fungi [10, 61], and other soil microbes [62, 63], it is often not included in EM enzymatic studies (but see refs. [64, 65]). Like APM, soil APD is integral to P cycling [15]. Phosphodiesters are considered to be important for the replenishment of P_i_ in soils because their phosphate groups are shielded from ionic interactions, making them less likely to be adsorbed to soil particles and more accessible to enzymatic cleavage [66, 67]. The activity of APD can be the rate-limiting step in P_o_ turnover of some soils [68] because, unlike the P in monoesters, the release of P from soil phosphodiesters appears to be limited by enzyme availability [16]. In our study, the net increase in APD activity was twice that of APM across the edaphic gradient, emphasizing the focused allocation of resources to this exoenzyme.

The strong relationship we uncovered between EM exoenzyme activity and foliar N:P ratio was a key finding of this study and illustrates succinctly how the mycorrhizas likely participated in an optimal allocation strategy [31]. According to this model, plants and mycorrhizal fungi allocate biomass and energy to strategies that will acquire the more limiting nutrient(s), because organisms function optimally when stoichiometry is maintained. Consequently, at N:P foliar ratios below a threshold (generally 14–16) [69], resources would be expected to be focused on N acquisition, but above this threshold the limitations from P would equal and then surpass that of N. In this way the energy devoted by the EM fungi to exoenzyme allocation was more precisely governed by P deficiency in relation to N constraints, rather than P availability alone. The N:P ratios of EM fruiting bodies from these plots were nearly identical to *P*. *menzesii* foliar N:P [7], so the exoenzyme allocation strategy documented here reflects the stoichiometry of both the fungal symbiont and the tree, as would be expected if the host/symbiont relationship is viewed as a “holobiont” [70]. Foliar N:P, it should be noted, is a more expedient measure of resource availability than soil pools, especially P_o_, but can also be a somewhat dynamic property that is affected by, for example, stand development stage [71] or interannual climatic variation [72].

The prominent EM fungal species producing the highest quantities of P-acquiring exoenzymes (e.g., *Lactarius substriatus*, *Russula brevipes* var. *acrior*, *Lactarius subviscidus*) were found only on the strongly podzolized soils of the west coast of Vancouver Island, both in the sampling of this study and in previous, extensive EM fungal surveys of eastern Vancouver Island [73]. The wholescale species turnover within EM fungal communities likely selected for species that are adapted to exploiting P_o_, as indicated by enzymatic traits, and thereby able to outcompete other EM and saprotrophic fungi [29, 74]. Because of the dependence of *P*. *menzesii* on ectomycorrhizas for acquiring soil nutrients, these traits would also maximize fitness of the host in response to P scarcity, with better adapted EM fungi possibly favored by the host tree through the targeted provision of carbon [75], or by downregulation of plant defenses during root colonization [76]. Through competition with other fungi and/or support by the tree, assembly of the Podzol EM fungal communities could have involved selection of either fungal species [77] or individual fungi within the population with appropriate P-acquisition traits, or acclimation of existing fungi to the low P_i_ and high P_o_ through upregulation of phosphatase genes. Given the high β diversity across this landscape, it appears less likely that capacity for physiological plasticity by fungal individuals or intraspecific variations were drivers of EM fungal community assembly here [27]. Some controlled, manipulative studies (e.g., reciprocal seedling transplants across soil types; [78]) could be used to test these capacities more thoroughly.

EM fungi are known to structure bacterial communities on their mantle surfaces and in soil [79]. The contribution of mantle-associated bacteria and archaea to exoenzyme activities measured in our study is not known, but incorporating antibiotics into assays have indicated a minor effect [80]. In addition, some of the variation in exoenzyme activities could reflect in part the morphology of the mantle, particularly for medium- and long-distance exploration types if extramatrical hyphae were lost during root preparation [44]. However, this potential bias should not have affected the average EM community response in exoenzyme activity since we found a mix of contact, short- and medium-distance mantles across all study plots. Furthermore, the considerable differences in exoenzyme activity among taxa within an exploration type, such as the contact mantle of *Russula xerampelina* and *Lactarius substriatus*, are consistent with a dominant effect of fungal species on these key traits [20–22, 41, 81].

## Conclusions

A podzolization gradient across a short transect of temperate coastal forests provided an ideal field test for differences in P-acquisition strategies among EM fungal communities of *P*. *menzesii*. Our results establish a significant role for APD in the acclimation of EM fungi to low P_i_ soils. The close link in exoenzyme activity with host stoichiometry highlighted the importance of assessing P deficiency in relation to N constraints, rather than P alone, as would be consistent with an optimal allocation strategy. The EM fungal species with the highest expression of key exoenzymes were found only on Podzols and underscore how resource constraints, such as sharp declines in P_i_ with soil podzolization, likely drive species turnover and the assembly of distinct, well-adapted EM fungal communities. Large increases in many key exoenzymes by site-specific EM fungi illustrate how a diverse symbiotic community greatly enhances the ability of a single tree host to accommodate the severe edaphic constraints inherent to these wet coastal landscapes.

## Supplementary information

Representative 31P-NMR spectra of forest floor for each soil type

31P-NMR spectroscopy protocol, EM root surface-bound exoenzyme assay, DNA extraction and PCR amplification of the fungal ITS region

Ectomycorrhizal fungal species distribution and frequency by soil type

## Data Availability

Available at Dryad (10.5061/dryad.tht76hdxq). Molecular sequences accessioned with UNITE (UDB0779903–UDB0779958).
