# Supplementary material for: Phosphorus deficiencies invoke optimal allocation of exoenzymes by ectomycorrhizas
Source: ISME J. 2021 Jan 8;15(5):1478–89. doi: 10.1038/s41396-020-00864-z (PMC8114911; doi:10.1038/s41396-020-00864-z)
Supplement: Supplementary file 1 — Representative 31P-NMR spectra of forest floor for each soil type [file 41396_2020_864_MOESM1_ESM.docx]

**Supplemental Figures. Representative ^31^P-NMR spectra of forest floor for each soil type**

**
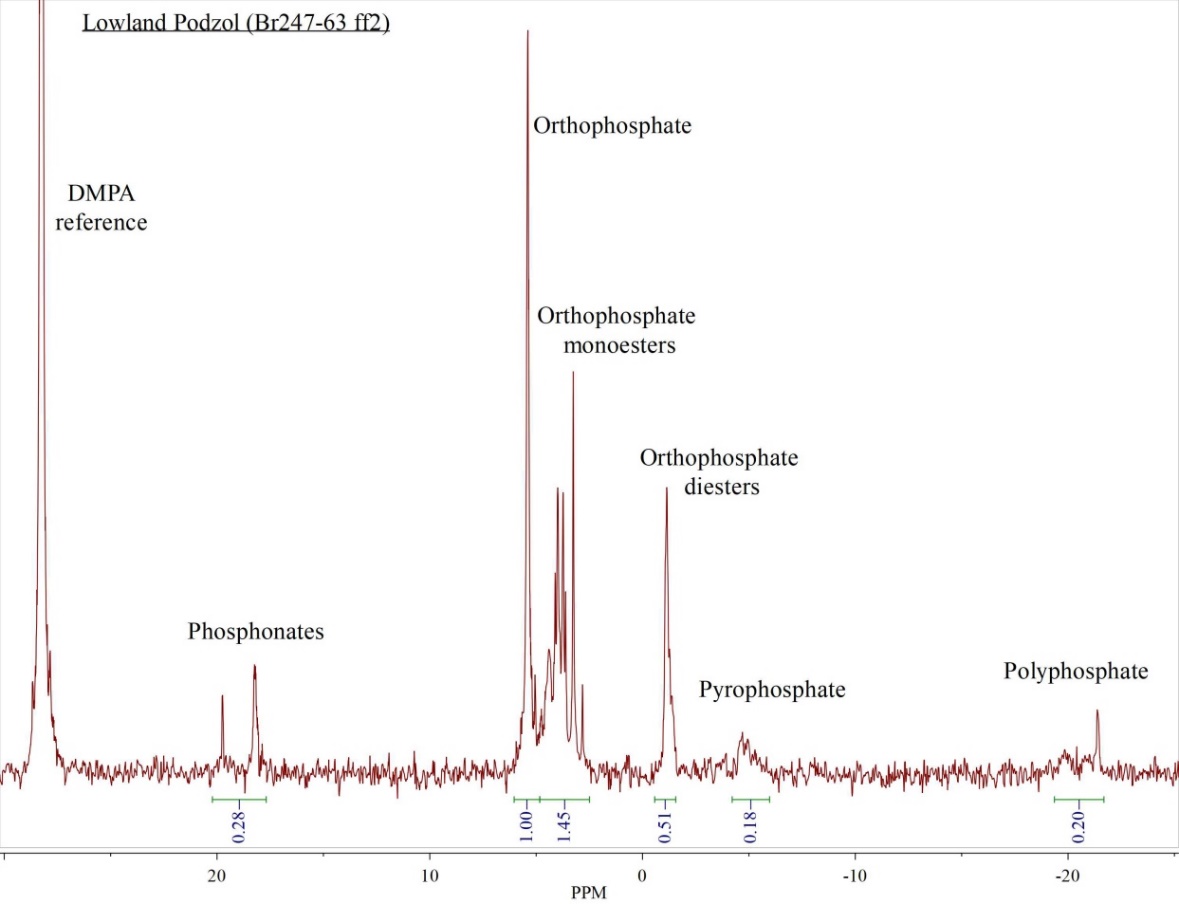
**

Supplemental Figure 1. ^31^P-NMR spectra for a lowland Podzol (Br 247-63)


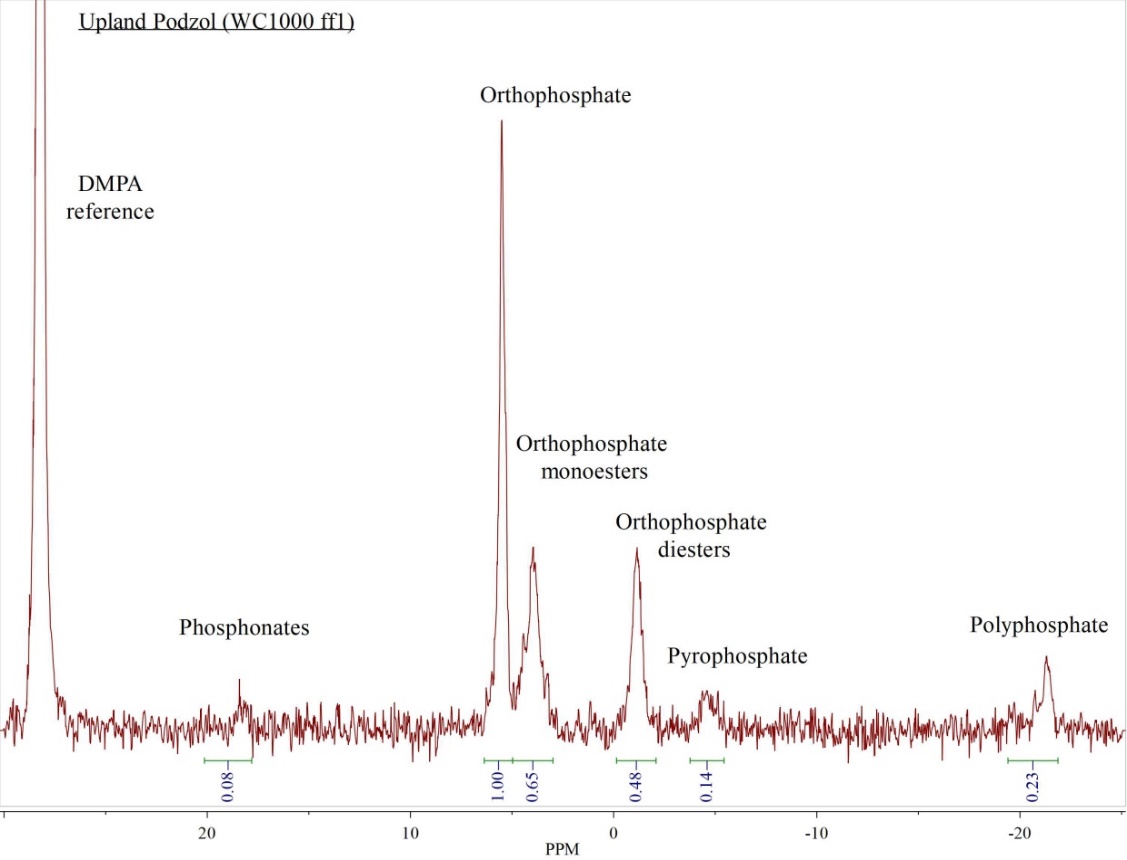


Supplemental Figure 2. ^31^P-NMR spectra for an upland Podzol (WC1000)


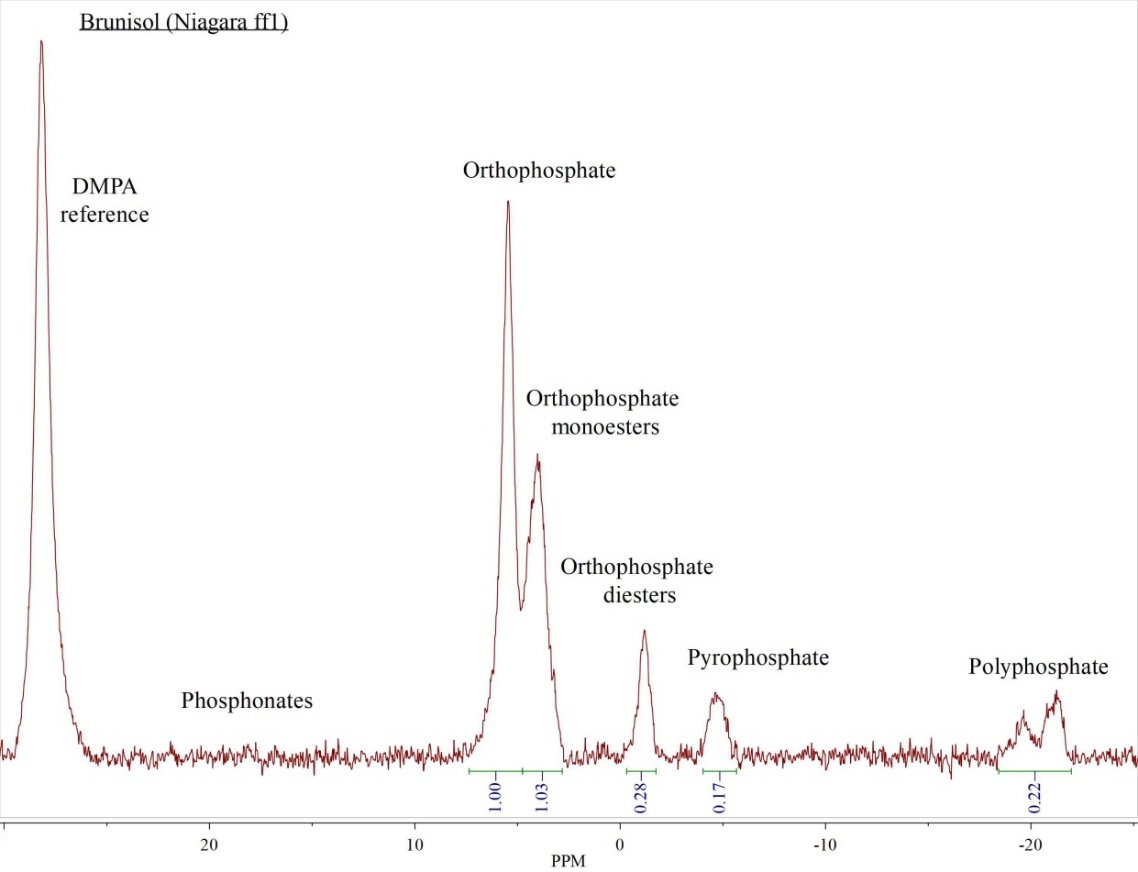


Supplemental Figure 3. ^31^P-NMR spectra for an upland Brunisol (Niagara)
