# Supplementary material for: Phosphorus deficiencies invoke optimal allocation of exoenzymes by ectomycorrhizas
Source: ISME J. 2021 Jan 8;15(5):1478–89. doi: 10.1038/s41396-020-00864-z (PMC8114911; doi:10.1038/s41396-020-00864-z)
Supplement: Supplementary file 2 — 31P-NMR spectroscopy protocol, EM root surface-bound exoenzyme assay, DNA extraction and PCR amplification of the fungal ITS region [file 41396_2020_864_MOESM2_ESM.docx]

Supplemental Method 1. **^31^P-NMR spectroscopy protocol**

NMR samples were prepared by shaking 3.0 g of air-dried soil in 25 ml of 0.5 M NaOH and 0.1 M disodium EDTA extracting solution for 4 hours. Extractions were centrifuged for 20 minutes at 3700 rpm, filtered, and the supernatant was freeze-dried. Dimethyl methyl phosphonic acid (DMPA; Sigma D169102) was used as an internal standard prepared by mixing 0.05 g of DMPA with 5.0 ml of the same extracting solution. NMR solvent was made by adding 0.8 ml of 10 M NaOH, 0.45 ml of internal standard, and 1.65 ml of deuterated water (D_2_O) to 1.5 ml of extracting solution (EDTA-NaOH). Samples were re-dissolved by adding the minimum amount of NMR solvent needed, starting with 2 ml and adding 0.5 ml at a time until fully dissolved, then vortexing intermittently for 5 minutes. Re-dissolved samples were centrifuged for 20 minutes at 4000 rpm, filtered through 8-10 µm ashless filter paper (Sartorius 389F), followed by filtration through 6 µm filter paper (Whatman, grade 3), and transferred to a 5 mm NMR tube. NMR analysis was performed as soon as possible after sample preparation; unused re-dissolved samples were stored at -4°C.

^31^P NMR spectra were acquired using a Varian Mercury VX400 spectrometer at 161.98 MHz. All experiments were performed at 25°C using a 90° pulse, a relaxation delay of 4.36 s, and an acquisition time of 0.61 s. Data was acquired until there was sufficient signal for integration (average number of scans 20,000). The peak of 85% orthophosphoric acid in D_2_O was used as an external reference at 0.00 ppm. Data was acquired from -25 to 30 ppm. This range includes chemical shifts for all environmentally relevant P compounds as assigned in the literature (1). DMPA was chosen as an internal standard because its chemical shift of 28.6 ppm (2) does not overlap with signals from any compounds of interest. Some pure standards, including DMPA, were measured in the NMR solvent to confirm the literature peak locations, and DMPA signal was tested over four days to ensure that the compound does not hydrolyze in the NMR solvent.

NMR spectra were processed using MestreNova v12.0 software. The time-domain data were apodized using a 2 Hz exponential multiplication prior to Fourier transformation. Peaks were assigned according to literature values (Cade-Menun 2005): free orthophosphate at ~6.0 ppm, orthophosphate monoesters at 3.0-7.0 ppm, orthophosphate diesters at -3 to +2.5 ppm, pyrophosphate at -5 ppm, polyphosphate middle groups at -20 ppm, polyphosphate end groups at -4 ppm, and phosphonates at 7-20 ppm. Only the phosphate groups in the middle of a polyphosphate chain were counted as polyphosphate; the signal from the end groups in the chain was not distinguishable from pyrophosphate signal. Linear regressions were calculated between the ratios of phosphomonoester and phosphodiester concentrations to orthophosphate concentrations obtained by NMR.

**References**

1. Cade-Menun BJ. Characterizing phosphorus in environmental and agricultural samples by ^31^P nuclear magnetic resonance spectroscopy. Talanta 2005;66:359–371.

2. Cade-Menun BJ. Improved peak identification in ^31^P-NMR spectra of environmental samples with a standardized method and peak library. Geoderma 2015; 257–258:102–114.

Supplemental Method 2. **EM root surface-bound exoenzyme assay**

All of the exoenzyme assays involve excised EM root-tips incubated in solutions containing the substrates that they act upon. Most of the substrates are linked to a fluorescent molecule, either methylumbelliferone (MU) or 7-amino-4-methylcoumarin (AMC), and result in a value of activity per area of root-tip (μmol min^-1^ mm^-2^) as measured by fluorescence of MU or AMC at 445 nm upon cleavage from the flourogenic substrate (1). Two of the enzymes (acid phosphodiesterase (APD), and laccase (LAC)) result in a colorimetric product, measured at various wavelengths (APD = 415nm, LAC = 420nm). Due to the pH sensitivity of the exoenzymes in question, all solutions were brought to the appropriate pH (either pH 4.5 or pH 6.5).

A 96-well filter plate was first filled with a rinsing buffer solution. Five EM roots from each colony, after cleaning, were placed into five individual buffer-filled wells of a 96-well filter plate, leaving at least 16 wells empty for a standard curve, substrate blanks, and buffer blanks. The EM roots were left stored in the buffer until the substrate working solutions were made. Once the working solutions, and the first few incubation solutions were made, the buffer in the filter plate was centrifuged out (10,000 rpm). At this point, using a multi-channel pipette, the first incubation solution was added to the filter plate. The EM roots were incubated for the appropriate amount of time, and the incubation solution was centrifuged into a black plate containing stopping buffer (Tris 1M, pH 10-11). The plate with the stopping buffer and incubation solution was then measured for fluorescence (364 +/- 10 nm excitation, and 445 +/- 15 nm emission) in a microplate reader. While the black plate was being measured, rinsing buffer was then added to the filter plate containing the EM roots. The protocol was repeated for each subsequent exoenzyme, using the same series of steps. For exoenzyme activity assays that resulted in a colorimetric product a clear 96-well plate was used to collect the incubation solution, and this plate was then measured for colour intensity (at 415 +/- 5 nm, or 420 +/- 5 nm, depending on the exoenzyme).

**APD assay**

The original protocol was for a soil enzyme assay (2). In this assay the same concentration was kept for the colorimetric substrate that the phosphodiesterase cleaves, and the volume of that solution per sample was reduced to microplate volumes, following the same series of steps as laid out in the EM root surface-bound exoenzyme protocol. The colorimetric substrate for the assay was bis-*p-*nitrophenyl phosphate (BPNP) (5 mM), which was made up fresh as a working solution each week. The calibration solution was made using *p*-nitrophenol (*p-NP)* (50 mM). All other solutions used in the assay were the same as were used in the EM root surface-bound exoenzyme protocol.

**Exoenzyme activity calculations**

Each individual assay resulted in a fluorescence (X, NAG, GU, & APM) or absorbance (APD & LAC) reading for each EM root in the 96-well plate. Fluorescence and colorimetric readings were measured using a Synergy HTX plate reader (BioTek Instruments, Winooski, VT, USA). From these readings, the enzyme activities were calculated (Eqn. 1) (3).

EA = $\frac{x-sub}{a \times pa \times t}\times\frac{{vol}_{tot}}{{vol}_{meas}}$ [1]

where $x$ is the measured value of the sample, *sub* is the measured value with substrate, but without sample, *pa* is the projection area of the mycorrhizal roots [mm^2^], *t* is the incubation time [min], vol_tot_ is the total volume of the incubation solution per well of an incubation plate, and vol_meas_ is the volume of the incubated solution that was finally measured.

For fluorescence measurements, *a* is the slope of the regression line of the calibration curve [μmol^-1^].

For the colorimetric measurements

*a =*$\varepsilon_{425}\times pl$ [2]

where ε_425_ is the molar coefficient of extinction for ABTS (ε_425_ = 3.6 x 10^4^ cm^2^ mol^-1^) and *pl* is the path length within the liquid of the well [cm].

Following the assays, the root tips were transferred into wells in a clear 96-well microplate containing 100 μL of water in each well. The plate was scanned using a high-resolution scanner (dpi 6400; Epson V800) and analysed using WinRhizo software (Regent Instruments Inc., Québec, Canada, 2017), allowing an estimate of the surface area to put into Eqn. 1.

**References**

1. Hoppe H-G. Significance of exoenzymatic activities in the ecology of brackish water: measurements by means of methylumbelliferyl-substrates. Marine Ecol. Prog. Series 1983;11: 299–308.

2. Eivazi F, Tabatabai MA. Phosphatases in soils. Soil Biol. Biochem. 1977;9:167-172.

3. Jones MD, Twieg BD, Ward V, Barker J, Durall DM, Simard SW. Functional complementarity of Douglas-fir ectomycorrhizas for extracellular enzyme activity after wildfire or clearcut logging. Func. Ecol. 2010;24:1139–1151.

Supplemental Method 3. **DNA extraction and PCR amplification of the fungal ITS region.**

Excised root tips from each collected morphotype were soaked in 200 µl of CTAB buffer for overnight incubation at 4°C. The next day samples were heated to 65°C for 30 minutes and mechanically ground with a micropestle (Kimble Kontes). Heating and grinding were repeated and an additional 300 µl of hot CTAB buffer was added to the suspension and incubated for another 60 minutes, followed by 1.5 µl of RNase A (94.3 Kunitz U/mg) at 37°C for 15 minutes. Next an equal volume of chloroform was mixed into the suspension and centrifuged at 13000 g for 10 minutes. The aqueous phase was transferred into a clean microcentrifuge tube and mixed with 0.08 volume of cold 7.5 M ammonium acetate, and 0.54 volume of cold isopropanol. The solution was incubated on ice for 30 minutes and centrifuged at 13000 g for 10 minutes. The supernatant was removed and 3 x washes of the DNA pellet were made with 700 µl of 70% EtOH. One ml 95% EtOH was added to the pellet, and left overnight at -20°C before centrifuging. Pellets were air dried and rehydrated with 15-40 µl TE buffer pH 8.0, depending on the size. One microliter of diluted DNA (10^-1^) was used as template for PCR amplification using Kappa HiFi polymerase (Roche Life Sciences) in a total reaction volume of 25 µl. The basidiomycete-specific ITS1F/LR21 or universal ITS5/ITS4 primer pairs were used to amplify the nuclear 5.8S rRNA gene and flanking internal transcribe spacers (ITS) (1,2). The final concentration of individual PCR reaction components were: 1X PCR buffer, 2.5 mM MgCl_2_, 200 µM each of dATP, dCTP, dGTP and dTTP, 1.0 units of Taq DNA polymerase per 25 µl reaction and 1 µM of each of the two primer pairs used. PCR amplification was carried out using a Applied Biosystems Veriti thermocycler (Thermo Fisher Scientific). Thermocycling conditions included an initial denaturation step of 95°C for 180 s followed by 35 amplification cycles of denaturation, annealing and extension. The temperature and times for these steps were 98°C for 20 s, 58°C for 30 s, and 72°c for 45 s. After the 35 cycles were completed the samples were incubated an additional 10 minutes at 72°C (2). Samples were then stored at 4°C.

Amplified PCR products were confirmed on a Qiaxcel Advanced capillary electrophoresis system equipped with standard DNA screening cartridge (Qiagen). Sequencing was performed at Genome sequencing and genotyping platform of the CHUL Medical Research Centre (Quebec, QC, Canada) using capillary electrophoresis on an ABI 3730 / XL Analyzer (Applied Biosystems). Forward and reverse sequences were aligned and manually corrected in Sequencher 5.4 (GeneCodes, Ann Arbor, MI, USA).

**References**

1. White TJ, Bruns T, Lee S, Taylor J. Amplification and direct sequencing of fungal ribosomal RNA genes for phylogenetics. In M.A. Innis, D.H. Gelfand, J.J. Sninsky, & T.J. White (Eds.), PCR Protocols: a Guide to Methods and Applications. Academic Press, New York; 1990. p. 315-322.

2. Gardes M, Bruns TD. ITS primers with enhanced specificity for basidiomycetes – applications to the identification of mycorrhizae and rusts. Mol. Ecol. 1993;2:113-118.
