# Supplementary material for: Phosphorus deficiencies invoke optimal allocation of exoenzymes by ectomycorrhizas
Source: ISME J. 2021 Jan 8;15(5):1478–89. doi: 10.1038/s41396-020-00864-z (PMC8114911; doi:10.1038/s41396-020-00864-z)
Supplement: Supplementary file 3 — Ectomycorrhizal fungal species distribution and frequency by soil type [file 41396_2020_864_MOESM3_ESM.docx]

Supplemental Table 1. Ectomycorrhizal fungal species distribution and frequency by soil type. Putative species name based on 98.5%-99.5% match with Species Hypothesis in UNITE.

| **Fungal taxa by soil type** | **Species Hypothesis** | **Frequency** |
| --- | --- | --- |
| **Upland Brunisols** |  |  |
| *Cenococcum geophilum* | SH1639590.08FU | 4 |
| *Clavulina* sp. | SH1505718.08FU | 1 |
| *Cortinarius* cf *acutus* | SH1503835.08FU | 1 |
| *Cortinarius casimiri* | SH1545222.08FU | 1 |
| *Cortinarius* sp. | SH1503874.08FU | 1 |
| *Elaphomyces* sp. | SH1587494.08FU | 1 |
| *Hebeloma* cf *crustuliniforme* | SH1563788.08FU | 1 |
| *Helvella vespertina* | SH1505149.08FU | 1 |
| *Hydnum* sp. | SH1539271.08FU | 1 |
| *Inocybe* sp. | SH1577177.08FU | 1 |
| *Inocbye* sp. | SH1529138.08FU | 1 |
| *Inocybe whitei* | SH1611227.08FU | 2 |
| *Laccaria* cf *bicolor* | SH1553002.08FU | 1 |
| *Lactarius rubrilacteus* | SH1726120.08FU | 3 |
| *Lactarius* sp. | SH1519114.08FU | 1 |
| *Piloderma byssinum* | SH1544810.08FU | 2 |
| *Piloderma olivaceum* 1 | SH1544814.08FU | 2 |
| *Piloderma olivaceum* 2 | SH1544805.08FU | 3 |
| *Piloderma* sp. | SH1544809.08FU | 3 |
| *Rhizopogon vesiculosus* | SH1630053.08FU | 3 |
| *Russula cascadensis* | SH1646311.08FU | 1 |
| *Russula nauseosa* | SH1625349.08FU | 2 |
| *Russula xerampelina* | SH1702028.08FU | 7 |
| Thelephoraceae sp. 1 | SH1502188.08FU | 1 |
| Thelephoraceae sp. 3 | N/A | 1 |
| Thelephoraceae sp. 4 | SH1502405.08FU | 1 |
| Thelephoraceae sp. 7 | SH1502442.08FU | 1 |
| *Tomentella* sp. 2 | SH1528512.08FU | 1 |
| *Tomentella* sp. 3 | SH1502196.08FU | 1 |
| *Tomentella* sp. 4 | SH1502208.08FU | 1 |
| *Tomentella terrestris* | SH1502202.08FU | 1 |
|  |  |  |
| **Upland Podzols** |  |  |
| *Amphinema byssoides* | SH1648320.08FU | 1 |
| Atheliaceae sp. | SH1529387.08FU | 1 |
| *Cenococcum geophilum* | SH1639590.08FU | 2 |
| *Clavulina* sp. | SH1560237.08FU | 1 |
| *Clavulina* cf *cristata* | SH1711054.08FU | 1 |
| *Cortinarius* sp. | SH1503874.08FU | 1 |
| *Cortinarius* sp. | N/A | 1 |
| *Cortinarius* cf *semisanguineus* | SH1855730.08FU | 2 |
| *Cortinarius* sp. | SH1503857.08FU | 1 |
| *Cortinarius malicorius* | SH1503720.08FU | 1 |
| *Inocybe* sp. | SH1562688.08FU | 1 |
| *Lactarius subviscidus* | SH1679722.08FU | 5 |
| *Lactarius substriatus* | SH1679693.08FU | 1 |
| *Piloderma byssinum* | SH1544810.08FU | 1 |
| *Pseudotomentella* sp. 1 | SH1656383.08FU | 1 |
| *Pseudotomentella* sp. 2 | SH1564296.08FU | 1 |
| *Rhizopogon parksii* | SH1630059.08FU | 1 |
| *Rhizopogon vesiculosus* | SH1630053.08FU | 2 |
| *Rhizopogon villosulus* | SH1630049.08FU | 1 |
| *Rhizopogon vinicolor* | SH1630051.08FU | 1 |
| *Russula* sp. | SH1509051.08FU | 1 |
| *Russula dissimulans* | SH2310089.08FU | 3 |
| *Russula veternosa* | SH1538877.08FU | 1 |
| *Sebacina* sp. 1 | SH1731043.08FU | 2 |
| *Sebacina* sp. 2 | SH1572357.08FU | 1 |
| *Sebacina* sp. 3 | SH1542779.08FU | 3 |
| Thelephoraceae sp. 6 | SH1502433.08FU | 1 |
| *Tomentella* sp. | N/A | 1 |
| *Tomentella* sp. | SH1659969.08FU | 2 |
| *Tomentellopsis* sp. | N/A | 1 |
| *Tylospora fibrillosa* | SH1964740.08FU | 1 |
|  |  |  |
| **Lowland Podzols** |  |  |
| *Amphinema* sp. | SH1648334.08FU | 2 |
| *Amphinema byssoides* | SH1648320.08FU | 3 |
| *Cenococcum geophilum* | SH1639590.08FU | 1 |
| *Cortinarius* sp. | SH1545401.08FU | 1 |
| *Cortinarius olivaceoluteus* | SH1503982.08FU | 1 |
| *Inocybe* cf *geophylla* | SH1611238.08FU | 1 |
| *Inocybe* cf *mixtilis* | SH1604101.08FU | 1 |
| *Inocybe* sp. | SH1529142.08FU | 1 |
| *Inocybe* sp. | SH1562688.08FU | 1 |
| *Lactarius subviscidus* | SH1679722.08FU | 3 |
| *Lactarius substriatus* | SH1679693.08FU | 3 |
| *Pseudotomentella* sp. | N/A | 1 |
| *Rhizopogon parksii* | SH1630059.08FU | 1 |
| *Rhizopogon vinicolor* | SH1630051.08FU | 1 |
| *Russula brevipes* var *acrior* | SH1865355.08FU | 3 |
| *Russula dissimulans* | SH2310089.08FU | 7 |
| *Sebacina* sp. | N/A | 1 |
| *Sebacina* sp. 3 | SH1542779.08FU | 1 |
| Thelephoraceae sp. 4 | SH1502405.08FU | 2 |
| Thelephoraceae sp. 5 | SH1853196.08FU | 2 |
| Thelephoraceae sp. 6 | SH1502433.08FU | 1 |
| Thelephoraceae sp. 8 | SH2714262.08FU | 1 |
| *Tomentella* sp. | SH1690920.08FU | 1 |
| *Tomentella* sp. | SH1659969.08FU | 1 |
| *Tomentella* sp. | SH1610990.08FU | 3 |
| *Tomentella sublilacina* | SH1502195.08FU | 1 |
| *Tricholoma atroviolaceum* | SH1517763.08FU | 1 |
| *Tylospora* sp. | SH1571573.08FU | 1 |
